# Correction: Shape anisotropy induced jamming of nanoparticles at liquid interfaces: a tensiometric study

**DOI:** 10.1039/d4na90090a

**Published:** 2024-08-14

**Authors:** Chandan Kumar, Suman Bhattacharjee, Sunita Srivastava

**Affiliations:** a Soft Matter and Nanomaterials Laboratory, Department of Physics, Indian Institute of Technology Bombay Mumbai 400 076 India sunita.srivastava@phy.iitb.ac.in +91-22-2576-7572; b Centre for Research in Nanotechnology & Science (CRNTS), Indian Institute of Technology Bombay Mumbai 400 076 India

## Abstract

Correction for ‘Shape anisotropy induced jamming of nanoparticles at liquid interfaces: a tensiometric study’ by Chandan Kumar *et al.*, *Nanoscale Adv.*, 2024, https://doi.org/10.1039/d4na00280f.

The authors regret the incorrect current affiliation address of Chandan Kumar was provided in the original manuscript. The correct affiliation address for Chandan Kumar is as follows: Institute of Materials and Systems for Sustainability (IMaSS), Nagoya University, Furo-cho, Chikusa-ku, Nagoya, Aichi, 464-8601, Japan, as can be seen in the affiliation details herein.

The Royal Society of Chemistry apologises for these errors and any consequent inconvenience to authors and readers.

